# Can Locomotor Performance Predict the Final Result of a Football Match? A Machine Learning Approach

**DOI:** 10.3390/s26113278

**Published:** 2026-05-22

**Authors:** Julen Castellano, Aitor Pinedo-Jauregi, Roberto Lopez del Campo, Ricardo Resta, Jesús Cámara

**Affiliations:** 1 GIKAFIT Research Group, Department of Physical Education and Sport, Faculty of Education and Sport, University of the Basque Country (UPV/EHU), 01007 Vitoria-Gasteiz, Spain; aitor.pinedo@ehu.eus (A.P.-J.); jesus.camara@ehu.eus (J.C.); 2 AKTIBOki—Research Group in Physical Activity, Physical Exercise and Sport, Department of Physical Education and Sports, Faculty of Education and Sport, University of the Basque Country (UPV/EHU), 01007 Vitoria-Gasteiz, Spain; 3 Department of Competitions and Mediacoach, LaLiga, 28043 Madrid, Spain; rlopez@laliga.es (R.L.d.C.); rresta@laliga.es (R.R.)

**Keywords:** soccer, match performance, time-motion, machine learning

## Abstract

The aim of this study was to predict the match outcome using locomotor-performance-related data from teams in both Spanish professional leagues. All matches from the first and second Spanish divisions (LaLiga and LaLiga2, respectively) across two consecutive seasons were used. The locomotor variables were as follows: total distance (TD) and distance covered at >21 km·h^−1^ (HSR), distinguishing between different game moments (in-possession, out-of-possession, and ball stopping). Match outcomes (win/lose) were predicted using a LASSO-regularized logistic regression based on standardized locomotor variables. Model performance was evaluated through accuracy, precision, recall, F1-scores, and AUC–ROC, demonstrating strong discriminative capacity and balanced classification across outcomes. The LASSO-regularized logistic regression model achieved strong predictive accuracy (76.8%) and balanced classification performance (F1 = 0.77; AUC = 0.85). TDnoPosmin, TD21posmin, TD21min, and TDoffmin emerged as key positive predictors of victory, whereas TD21noPosmin, TDmin, and TDposmin were negatively associated with winning. LASSO regularization confirmed the stability and robustness of these predictors, indicating limited overfitting and consistency. Match outcomes were accurately predicted from locomotor variables, with high-intensity activity out of possession emerging as the key determinant of success. Match success was primarily linked to high-intensity activity during the defensive phase, highlighting the need for further research on these critical phases of play.

## 1. Introduction

Today’s world is marked by an unprecedented proliferation of data across nearly all sectors of society. From a scientific perspective, access to these datasets offers an exceptional opportunity for artificial intelligence (AI) and its derivative machine learning (ML) to develop, test, and implement new methods in real-world settings [1]. The sports domain has not been exempt from this evolution, and in football, AI and ML have been increasingly applied to match activity and analytics, talent identification, training and coaching, and rehabilitation and injury prediction [2,3].

In high-performance football, this data proliferation poses a significant analytical challenge: the effective management and interpretation of the vast volumes of information generated during matches and training sessions. Modern tracking systems and GNSS (Global Navigation Satellite System) technologies provide detailed spatiotemporal trajectories of players and the ball, alongside extensive event-based data. As highlighted by Memmert [4], nonlinear ML techniques offer a promising avenue to address this complexity and extract meaningful performance insights from multidimensional datasets. However, the value of these approaches depends not only on their predictive accuracy but also on their capacity to represent football performance as an integrated, dynamic, and context-dependent phenomenon rather than as the isolated expression of a single performance dimension.

Machine learning encompasses a broad range of supervised and unsupervised methods capable of addressing regression, classification, clustering, feature selection, and representation learning tasks, including advanced approaches based on neural networks and reinforcement learning [3]. In football research, ML has been widely adopted for match-outcome prediction, tactical analysis, player profiling, and injury-risk management. Match outcome modelling has attracted substantial attention. Algorithms such as neural networks and gradient-boosted trees have shown promising predictive performance. They achieve this by integrating historical performance indicators and contextual variables such as match location, team form, and previous results [5,6]. However, these approaches typically treat match performance as a consequence of historical trends rather than as a primary source of predictive information.

Despite these advances, a key limitation persists: Most predictive models rely predominantly on historical and contextual data, which restricts their capacity to reflect the dynamic nature of the match being played [6,7,8]. In contrast, alternative approaches have demonstrated the value of incorporating match-specific performance information. For instance, Fernández [9] proposed the Expected Possession Value (EPV) framework to quantify tactical decision-making during possession, Bojinov and Bornn [10] showed how pressing behaviours shape defensive effectiveness through spatial modelling, and Power et al. [11] highlighted how set-piece characteristics significantly influence scoring probabilities. These studies collectively indicate that performance variables generated during the match can offer explanatory and predictive value that is not captured by pre-match or historical descriptors alone. Importantly, these in-match indicators should not be understood as independent or causal determinants of success, but as partial expressions of the tactical, technical, emotional, physical, and contextual interactions that shape team behaviour during competition.

Nevertheless, several methodological challenges remain. Many ML approaches face a trade-off between predictive accuracy and interpretability, limiting their practical usefulness for coaches and practitioners. Moreover, predictive models often prioritize contextual descriptors (e.g., venue, weather, or team history), while variables intrinsic to competitive performance remain comparatively underexplored. Football performance is inherently multifactorial, emerging from the interaction of physical, technical–behavioural, tactical–decisional, psychological–emotional, and situational dimensions [12,13]. From a methodological standpoint, this underscores the need for models that can integrate high-resolution physical performance data with outcome prediction while maintaining interpretability and applicability in applied settings [5]. Accordingly, the study of locomotor variables should be framed within this broader ecological perspective: running demands do not explain match success in isolation, but may provide useful information about how teams respond physically to specific tactical and contextual constraints.

Although physical performance–related variables have been extensively examined to differentiate winning and losing teams [14] and to assess the influence of contextual factors on physical output [15], recent work predicting teams’ running demands as a function of opponents’ physical behaviour [16] opens a new avenue for match outcome prediction grounded in locomotor performance. Building on this perspective, the present study adopts a machine learning–based approach that leverages team-level locomotor performance indicators collected during matches to directly predict competitive outcomes. This approach does not assume that locomotor performance is the central cause of match outcome, but rather explores whether selected locomotor indicators, as observable manifestations of team behaviour, contribute to discriminating between wins and losses within the multidimensional nature of football performance. Therefore, the aim of this study was to assess the ability of locomotor performance variables to predict match outcomes (win vs. loss) across Spanish professional leagues (LaLiga and LaLiga2). This approach emphasizes in-match physical performance as a central predictive factor rather than as a secondary explanatory variable. It was hypothesized that teams exhibiting higher locomotor performance indicators would be more likely to win matches in both competitions. This approach considers in-match physical performance as one informative dimension of competitive behaviour, while acknowledging that match outcome emerges from the interaction of tactical, technical, physical, psychological, and contextual factors. It was hypothesized that selected locomotor performance indicators would contribute to discriminating between winning and losing teams in both competitions.

## 2. Methods

### 2.1. Participants

Performance from two consecutive seasons (2021–2022, *n* = 1534, and 2022–2023, *n* = 1636) across the two professional leagues in Spain (LaLiga, *n* = 1454, and LaLiga2, *n* = 1716) was analyzed, resulting in a total of 3170 team performances (94% of all possible performances). According to Lakens [17], a valid approach to sample size justification is to collect data from nearly the entire population, provided that the population is finite and that measuring almost all units is feasible. The Ethics Committee for research with humans (CEISH) of the University of the Basque Country (UPV/EHU) granted institutional approval for the study (code M10-2024-124).

### 2.2. Predictors and Predicted Variables

The predicted variable was the match outcome, defined as a binary categorical variable with two possible results: win or loss. Draws were excluded from the analysis because they may represent heterogeneous and less clearly differentiated competitive scenarios. Their inclusion as a third category would have reduced model parsimony and complicated the practical interpretation of the locomotor indicators associated with clearly successful or unsuccessful match outcomes for coaches and analysts. The predictor locomotor variables included locomotor performance metrics (Table 1). In line with previous studies [14,15], the variables were divided into two phases of the game: in-possession and out-of-possession phases.

### 2.3. Procedures

Physical data was obtained by the computerized multi-camera tracking system TRACAB^®^ Gen5 (TRACAB, EA Sports, Redwood City, CA, USA), and the effective playing time, pause time and possession data was obtained by the data company OPTA^®^ (Opta Sports, London, UK), both using Mediacoach^®^ technological ecosystem. The reports were generated using Mediacoach^®^ for the predefined performance indicators. The reliability of the OPTA^®^ system has been previously proved [18] and the reliability of the multi-camera tracking system TRACAB^®^ has also been tested for positioning and physical performance of the players [19]. The generated reports were exported into a Microsoft Excel spreadsheet (Microsoft Corporation, Redmond, WA, USA) to configure a matrix.

### 2.4. Data Analysis

A statistical analysis was conducted to model the probability of match outcomes (win or lose) using a binomial logistic regression. All predictor variables were standardized to ensure comparability and improve model convergence, and the dataset was split into training and testing sets using a 70/30 ratio. The *variance inflation factor* (*VIF*) test was first employed to quantify the degree of multicollinearity among predictors. Variables with *VIF* values exceeding 10 were considered to indicate high multicollinearity [20]. To enhance model parsimony and prevent overfitting, L1-regularized logistic regression (Lasso) was applied, leveraging its ability to perform feature selection by shrinking the coefficients of irrelevant predictors to zero [21].

**Figure 1:**
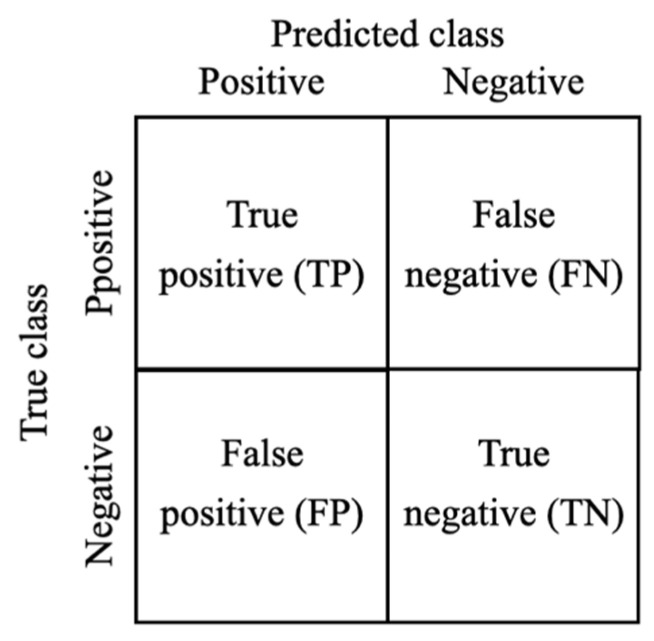
Performance measurements calculated on the basis of the confusion matrix.

Given that this study addresses a binary classification problem (win vs. loss), model performance was evaluated using a confusion matrix for positive and negative classes [22] as illustrated in Figure 1.

Primary metrics were calculated based on the confusion matrix: Accuracy, Precision, Recall, and F1-score. Accuracy represents the overall proportion of correct classifications (both wins and losses) among the total number of instances (Equation (1)). Precision (Equation (2)) indicates the model’s reliability in predicting the positive class (wins), while Recall (Equation (3)) measures the model’s ability to identify all actual positive cases. Finally, the F1-score (Equation (4)) is calculated as the harmonic mean of precision and recall, providing a balanced assessment that accounts for the trade-off between both metrics.

Equation (1). Accuracy:

(1) $$Accuracy=\frac{TP+TN}{TP+TN+FP+FN}$$

Equation (2). Precision:

(2) $$Precision=\frac{TP}{TP+FP}$$

Equation (3). Recall:

(3) $$Recall=\frac{TP}{TP+FN}$$

Equation (4). F1-Score:

(4) $$F1\text{-}Score=2*\frac{Precision*Recall}{Precision+Recall}$$

Additionally, the *area under the curve* (*ROC*) of the receiver operating characteristic (*ROC* = graph) was calculated to assess the model’s discriminative ability between the two outcome classes (win vs. loss). The *ROC* curve was plotted to visualize the trade-off between true positive rate and false positive rate across different classification thresholds, providing insight into the model’s performance across various decision boundaries. All the analyses were performed in Python (v3.12.7). Pandas was utilized for data management, scikit-learn for model construction and validation, and stats models for VIF diagnostics. Graphical representations were produced using seaborn and matplotlib.

## 3. Results

The VIF analysis identified multicollinearity in several variables (Table 2). Consequently, LASSO regularization was applied to confirm the stability of the most relevant predictors while mitigating redundancy and enhancing model parsimony. LASSO coefficients (|β|) were used to quantify variable importance and identify the most predictive variables.

The analysis of coefficients highlighted differences in predictive power among the variables (Table 2). *TDnoPosmin* emerged as the most influential factor associated with victory, reinforcing its central role in determining match outcomes. Other variables contributing to winning predictions included *TD21min*, *TD21posmin*, and *TDoffmin*, each showing meaningful positive effects. Conversely, *TD21noPosmin* was the strongest predictor of defeat, followed by *TDmin*, *TDposmin*, and *TD21emin*, which consistently showed negative associations with winning. In contrast, *TD21offmin* displayed only marginal influence, underscoring its limited predictive value.

Finally, the comparison between the baseline and LASSO-regularized models revealed that the coefficients remained practically unchanged. Although several variables exhibited high multicollinearity (*VIF* > 10, with *TD21min* reaching 102.7), the application of LASSO regularization effectively mitigated the potential instability inherent to these predictors. Unlike standard logistic regression, which often fails under severe multicollinearity due to inflated standard errors, LASSO manages redundant information by shrinking coefficients through an L1 penalty and performing automated feature selection. This ensures that the reported predictors are stable and not artifacts of variance inflation. The fact that the predictive hierarchy remained consistent after regularization (Table) indicates that LASSO successfully identified the unique contribution of each locomotor metric, filtering out the noise generated by high inter-variable correlations. This robustness is further supported by the high discriminative power achieved (AUC = 0.85; F1 = 0.77), confirming that the model’s stability and generalizability were not compromised by the internal redundancy of the data. Consequently, the LASSO model reinforced the robustness of the most powerful predictors for both victory (*TDnoPosmin*, *TD21posmin*, and *TDoffmin*) and defeat (*TD21noPosmin*, *TDmin*, and *TDposmin*).

Beyond the stability of influential predictors, the LASSO-regularized logistic regression model demonstrated solid predictive performance. The overall classification accuracy reached 76.8%, with balanced performance across classes. As shown in the confusion matrix (Table 3), the model correctly classified 254 defeats and 260 victories, while misclassifying 86 (25.3%) defeats as wins and 69 (21.0%) wins as defeats.

The classification performance of the LASSO-regularized logistic regression model demonstrated high consistency and discriminative reliability across both outcome classes (Table 4). The identical *F*1-score of 0.77 for victories and defeats, coupled with the narrow 95% confidence intervals obtained through bootstrapping, confirms that the model’s predictive ability is stable and not biassed toward either class. These bootstrapped intervals reinforce the robustness of the identified locomotor signatures, indicating that the high discriminatory power is consistent across different data subsamples.

**Figure 2:**
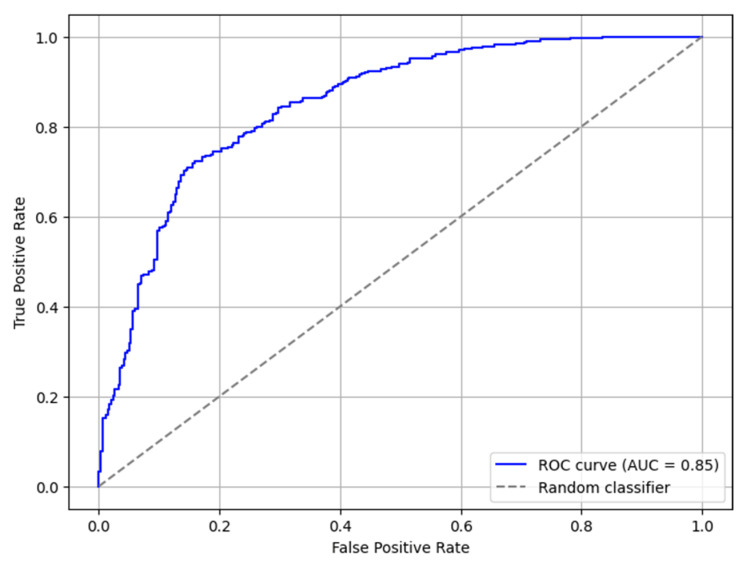
ROC curve and AUC for the Lasso-regularized logistic regression model.

The Receiver Operating Characteristic (ROC) graph provided a graphical assessment of the model’s discriminative ability. As illustrated in Figure 2, the Lasso-regularized logistic regression model achieved an Area Under the Curve (AUC) of 0.85, a value generally interpreted as very good discrimination (e.g., [23]). This suggests that the model effectively distinguishes between wins and losses while maintaining parsimony. Furthermore, the high AUC value confirms that the feature selection process inherent to the L1 penalty did not compromise the model’s predictive accuracy but rather enhanced its generalizability.

The *ROC* curve consistently lies well above the diagonal reference line, which represents random classification, indicating that the model distinguishes victories from defeats with a high degree of reliability. Specifically, an *AUC* of 0.85 indicates that in 85% of randomly chosen pairs consisting of one victory and one defeat, the model assigns a higher predicted probability to the victory. Moreover, the steep initial rise in the curve reflects a strong true positive rate at relatively low false positive rates, confirming that the model achieves good sensitivity without sacrificing much specificity.

Together with the balanced precision, recall, and F1-scores reported earlier, the *ROC* analysis confirms that the Lasso model not only preserved the most relevant predictors but also achieved robust predictive performance across different decision thresholds.

## 4. Discussion

The aim of the present study was to evaluate the predictive capacity of various locomotor performance variables regarding football match outcomes (win vs. loss) through a binomial logistic regression. While the results revealed high multicollinearity among predictors, the similarity between the *β* and *β*-*Lasso* coefficients suggests that the regularization process did not eliminate any variables but rather refined the magnitudes of highly collinear predictors. Specifically, the model stabilized estimates of *TD21min* and *TDmin*, allowing for a more robust interpretation of their predictive weights. Consequently, the model demonstrated high stability and consistent performance (*F*1-*score*: 0.77; *AUC* of 0.85).

From a tactical perspective, the findings suggest that match success is strongly linked to defensive volume and offensive intensity. The strong positive impact of *TDnoPosmin* (total distance per minute out-of-possession) indicates that winning teams cover a greater volume while defending. This is complemented by the importance of *TD21posmin* (high-intensity running in-possession), highlighting that when a team has the ball, the key to success lies in the ability to perform explosive efforts (>21 km/h) rather than maintaining mere possession.

Conversely, the model identifies specific performance profiles associated with defeat, such as the strong negative association of *TD21noPosmin* (high-intensity running out-of-possession). This suggests that high-intensity efforts while defending may be an indicator of defensive disorganization, forcing players into high-speed running to recover their positions as quickly as possible or to pursue opponents. These findings are consistent with [24], who observed that defensive players execute more out-of-possession high-intensity runs when the opponent’s goal probability is high. Furthermore, in our study, the negative coefficients for *TDmin* (total distance) and *TDposmin* (distance in-possession) reinforce the idea that excessive running volume, especially while in-possession, does not guarantee success and may instead reflect an ineffective, slow build-up or a lack of verticality in the team’s play.

While he found no correlations between team ranking and peak speed in competition [25], it seems that a high tempo in the defensive phase and a high-speed accumulation of distance in the offensive phase could differentiate the locomotor performance of teams that manage to win in matches [14].

Supporting this interpretation, the LASSO-regularized model demonstrated high discriminatory power in distinguishing between match outcomes, underscoring the relevance of the key predictors, particularly *TD21posmin* and *TDnoPosmin*. Given that several predictors showed high *VIF* values, indicating substantial multicollinearity, LASSO regularization was specifically implemented to reduce instability in coefficient estimates and select the most informative variables among correlated predictors. Although LASSO does not remove multicollinearity from the original data structure, it mitigates its influence by shrinking less relevant or redundant coefficients towards zero, thereby improving model parsimony, interpretability, and generalization [21]. The model achieved an overall accuracy of 76.8%, correctly identifying 79.0% of victories and 74.7% of defeats. These balanced classification rates suggest that the retained locomotor performance variables capture relevant information associated with winning and losing, providing a useful framework for predicting competitive success in professional football.

The symmetrical values observed across precision, recall, and *F*1-*scores* (0.77 for both outcomes) underscore the model’s ability to minimize prediction bias, ensuring equitable classification performance for both victories and defeats. Such a balance suggests that the identified locomotor metrics serve as highly discriminant features, capturing the physiological and tactical nuances in both successful and unsuccessful match scenarios with comparable efficacy. These findings align with earlier evidence showing that locomotor and intensity-based metrics (e.g., total distance, sprint distance, and high-speed running) are moderately related to team performance [14,15,16]. Nevertheless, while these findings validate the independent efficacy of locomotor data, previous research suggests that incorporating contextual and tactical variables could further refine predictive accuracy by accounting for additional sources of match variability [26,27].

Discriminative analysis yielded an *AUC* of 0.85, confirming that it correctly assigned higher winning probabilities in approximately 85% of randomly selected win–loss pairs. The steep rise in the *ROC* curve illustrates that the model achieved strong sensitivity at relatively low false-positive rates, indicating effective classification thresholds. According to Hosmer et al. [28], *AUC* values above 0.80 denote very good discrimination, supporting the conclusion that the selected predictors meaningfully capture differences between match outcomes. These findings validate the predictive relevance and robustness of the locomotor variables, reflecting a practical capacity to detect successful performances with minimal misclassification—consistent with other machine-learning applications in football that integrate multivariate physical profiles [29].

Nevertheless, the remaining misclassifications underscore the multifactorial nature of football performance [30], where decisional, energetic, affective, and behavioural dimensions, alongside contextual and situational variables, may also exert a decisive influence on competitive outcomes [5]. Generally, match outcomes in sports characterized by high competitiveness, low-scoring, and fewer scoring opportunities are inherently more difficult to predict [5]. Therefore, future research should incorporate broader, multidimensional feature sets that integrate diverse levels of analysis and varied information sources to capture the complexities affecting match results.

This study is not exempt from limitations. Firstly, the analysis focused exclusively on locomotor variables, omitting technical and tactical indicators that could enhance the model’s predictive capacity. Secondly, contextual and situational variables were not included, which would likely have further refined the accuracy of the results. Thirdly, the model was restricted to a binary classification approach by comparing wins and losses, while draws were excluded from the analysis. Although draws represent a frequent and relevant outcome in football, this decision was made to focus on clearly differentiated match outcomes. Draws may reflect heterogeneous competitive scenarios and could therefore introduce additional ambiguity into the interpretation of locomotor indicators associated with match success. Thus, the binary win–loss approach was adopted as an initial modelling strategy to examine whether locomotor performance variables could discriminate between clearly successful and unsuccessful outcomes in a low-scoring and inherently unpredictable sport such as football [5]. Future studies should consider draws as an independent outcome category or use multinomial or ordinal classification frameworks. Fourthly, although Lasso regularization was selected to improve model interpretability and reduce the influence of correlated predictors, this approach may not fully capture the nonlinear and interactive nature of football performance. Therefore, future research should compare Lasso-based models with alternative nonlinear machine learning approaches, such as Random Forests, Gradient Boosting, or Neural Networks, to better assess the robustness and methodological adequacy of predictive models in this context. Finally, while the dataset encompassed the majority of professional match performances in the Spanish league over two consecutive seasons, caution is warranted when extrapolating these findings to other leagues and seasons.

## 5. Conclusions

The findings suggest that selected locomotor performance indicators may help discriminate between wins and losses in professional football within a binary classification framework. Distances covered out-of-possession (*TDnoPosmin*), distance out-of-play (*TDoffmin*), and high-intensity efforts in-possession (*TD21posmin*, *TD21min*, *TDoffmin*) emerged as relevant predictors of victory. Conversely, total distance and high-speed running during prolonged possession phases were negatively associated with victory. The consistency of the selected predictors across baseline and LASSO-regularized models supports the relevance of these relationships, indicating that certain locomotor demands may help differentiate between winning and losing performances. However, these findings should be interpreted in light of the exclusion of draws, the absence of contextual variables, and the presence of multicollinearity among some predictors. Therefore, the results should be considered an initial step towards understanding the predictive value of locomotor performance in match outcome, rather than definitive evidence that physical performance alone can predict competitive success.

Accordingly, the present study does not assume a direct causal relationship between locomotor performance and match outcome. Instead, it adopts a predictive and exploratory perspective, examining whether selected locomotor indicators provide useful information to discriminate between wins and losses within the inherently multifactorial nature of football performance. From this perspective, an important next step would be to identify the tactical behaviours and game situations in which high-speed running actions occur and determine how these behaviours may contribute to increasing teams’ probability of competitive success.

## 6. Practical Applications

From a practical perspective, these results suggest that match success is more strongly associated with high-intensity actions during the in-possession phase and with a higher overall running tempo during the out-of-possession phase. During possession, players are likely required to create passing options or break the opponent’s lines through high-speed movements, whereas the initial moments of the defensive transition, immediately after a ball loss, appear especially decisive, as teams strive to recover possession or delay the opponent’s attack.

However, it must be acknowledged that the use of a Lasso-regularized logistic regression assumes a linear and additive relationship between locomotor variables and match outcomes. While this approach effectively handles the high multicollinearity observed in high-intensity metrics and provides a stable hierarchy of predictors, it may not fully capture the complex, non-linear interactions inherent to football performance.

As performance emerges from the dynamic interaction of physical and tactical variables, future studies should explore this aspect in greater detail to determine whether these intense defensive actions are indeed concentrated in the early phase of transition play and how they influence match outcomes.

## Figures and Tables

**Table 1:** Predictor and outcome variables of the Machine Learning Model.

Dimension	Variable	Definition
Locomotor variables	*TDmin*	Total distance (TD) of the team per min
	*TDnoPosmin*	TD of the team per min out-of-possession
	*TDposmin*	TD of the team per min in possession
	*TDoffmin*	TD of the team per min when the game is stopped
	*TD21min*	TD at >21 km/h of the team per min
	*TD21emin*	TD at >21 km/h of the team per min with the ball in play
	*TD21posmin*	TD at >21 km/h of the team per min in possession
	*TD21noPosmin*	TD at >21 km/h of the team per min out-of-possession
	*TD21offmin*	TD at >21 km/h per min when the game is stopped
Outcome	Score	Final result of the match: win (1) or lose (0)

**Table 2:** Variance inflation factor (VIF), baseline and LASSO (β) coefficients for predictors of match outcomes.

Variable	VIF	Baseline Coefficient (β)	LASSO Coefficient (β Lasso)
*TDnoPosmin*	13.8	1.63	1.67
*TD21posmin*	15.4	1.13	1.07
*TD21min*	102.7	1.11	1.19
*TDoffmin*	9.3	0.86	0.88
*TD21offmin*	1.9	0.72	0.71
*TD21emin*	59.5	−0.76	−0.76
*TDmin*	55.8	−0.89	−0.94
*TDposmin*	12.1	−0.94	−0.93
*TD21noPosmin*	22.3	−1.49	−1.56

Note: *TDmin* = total distance (TD) of the team per minute; *TDnoPosmin* = TD per minute out-of-possession; *TDposmin* = TD per minute in-possession; *TDoffmin* = TD per minute when the game is stopped; *TD21min* = TD per minute at >21 km·h^−1^; *TD21emin* = TD per minute at >21 km·h^−1^ with the ball in play; *TD21posmin* = TD per minute at >21 km·h^−1^ in-possession; *TD21noPosmin* = TD per minute at >21 km·h^−1^ out-of-possession; *TD21offmin* = TD per minute at >21 km·h^−1^ when the game is stopped.

**Table 3:** Confusion matrix of the Lasso-regularized logistic regression model.

	Predicted Negative (Defeat)	Predicted Positive (Win)
*Actual Negative* (*Defeat*)	254 (*TN*)	86 (*FP*)
*Actual Positive* (*Win*)	69 (*FN*)	260 (*TP*)

TN: True Negatives; FP: False Positives; FN: False Negatives; TP: True Positives.

**Table 4:** Classification performance of the Lasso-regularized logistic regression model with 95% confidence intervals (CI).

Outcome	Precision	Precision 95% CI	Recall	Recall 95% CI	F1-Score	F1-Score 95% CI
*Defeats*	0.79	[0.75, 0.83]	0.75	[0.72, 0.80]	0.77	[0.74, 0.80]
*Victories*	0.75	[0.72, 0.80]	0.79	[0.75, 0.83]	0.77	[0.75, 0.80]

CI: Confidence intervals.

## Data Availability

The original contributions presented in this study are included in the article. Further inquiries can be directed to the corresponding author.
